# Subcellular localization of the antidepressant-sensitive norepinephrine transporter

**DOI:** 10.1186/1471-2202-10-65

**Published:** 2009-06-23

**Authors:** Heinrich JG Matthies, Qiao Han, Angela Shields, Jane Wright, Jessica L Moore, Danny G Winder, Aurelio Galli, Randy D Blakely

**Affiliations:** 1 Department of Molecular Physiology and Biophysics, Vanderbilt University School of Medicine, Nashville, TN 37232, USA; 2 Department of Pharmacology, Vanderbilt University School of Medicine, Nashvillex, TN 37232, USA; 3 Center for Molecular Neuroscience, Vanderbilt University School of Medicine, Nashville, TN 37232, USA; 4 Department of Psychiatry, Vanderbilt University School of Medicine, Nashville, TN 37232, USA

## Abstract

**Background:**

Reuptake of synaptic norepinephrine (NE) via the antidepressant-sensitive NE transporter (NET) supports efficient noradrenergic signaling and presynaptic NE homeostasis. Limited, and somewhat contradictory, information currently describes the axonal transport and localization of NET in neurons.

**Results:**

We elucidate NET localization in brain and superior cervical ganglion (SCG) neurons, aided by a new NET monoclonal antibody, subcellular immunoisolation techniques and quantitative immunofluorescence approaches. We present evidence that axonal NET extensively colocalizes with syntaxin 1A, and to a limited degree with SCAMP2 and synaptophysin. Intracellular NET in SCG axons and boutons also quantitatively segregates from the vesicular monoamine transporter 2 (VMAT2), findings corroborated by organelle isolation studies. At the surface of SCG boutons, NET resides in both lipid raft and non-lipid raft subdomains and colocalizes with syntaxin 1A.

**Conclusion:**

Our findings support the hypothesis that SCG NET is segregated prior to transport from the cell body from proteins comprising large dense core vesicles. Once localized to presynaptic boutons, NET does not recycle via VMAT2-positive, small dense core vesicles. Finally, once NET reaches presynaptic plasma membranes, the transporter localizes to syntaxin 1A-rich plasma membrane domains, with a portion found in cholera toxin-demarcated lipid rafts. Our findings indicate that activity-dependent insertion of NET into the SCG plasma membrane derives from vesicles distinct from those that deliver NE. Moreover, NET is localized in presynaptic membranes in a manner that can take advantage of regulatory processes targeting lipid raft subdomains.

## Background

The neurotransmitter norepinephrine (NE) is synthesized, stored, and released by a distributed collection of neurons in the brainstem, by neurons of the sympathetic branch of the autonomic nervous system and by endocrine cells of the adrenal medulla [1-3]. The powerful and widespread actions of NE include the regulation of metabolism, cardiovascular function, memory, emotion, attention, arousal, and appetite [4-7]. These actions are supported by the plasmalemmal NE transporter (NET) [8,9] an integral membrane protein that binds and clears NE following release [10-12]. NET is a clinically important drug target, particularly for the treatment of mood and cognitive disorders, including depression and attention-deficit hyperactivity disorder [12,13]. NET is also targeted by psychostimulants, including cocaine and amphetamines [9,14-16]. NET dysfunction has been linked to attention, mood and cardiovascular disorders [17-25]. The importance of NET for normative physiology and behavior has been amply confirmed with studies of NET knock out (KO) mice that display altered seizure susceptibility and opiate/cocaine sensitivities, as well as maladaptive responses to social and cardiovascular stressors [26-33].

Given the powerful control exerted by NET over NE signaling, it is not surprising that the NET protein itself has been found to be highly regulated [12,34]. Changes in NET distribution and/or activity arise through the acute engagement of signaling pathways, that in turn may support the actions of psychostimulants [16,35]) as well as chronic stressors [36]. NET regulatory pathways impact both transporter surface trafficking and catalytic activity and are supported by multiple Ser/Thr and Tyr kinases [37-39] and phosphatases [40], as well as interacting proteins [41-43]. NET regulation appears to be supported by interactions with multiple associated proteins. Thus, NET has been shown to physically associate with PP2Ac, syntaxin 1A, Hic-5, PICK-1, 14-3-3 proteins, and α-synuclein [40,41,43-45]. How these interactions are coordinated is unclear. One possibility is that membrane subdomains may serve as a site at which distinct NET associations are acquired or stabilized. For example, in placental trophoblasts, NET localizes, in part, to lipid raft-containing membrane subdomains [46,47]. Surprisingly, similar data are currently lacking for neuronal NET.

In neuronal preparations and endocrine cell lines, NETs localize to both the plasma membrane as well as to intracellular vesicles, consistent with either biosynthetic transport vesicles or recycling compartments [36,48-50]. In the brain, NET surface expression in axonal varicosities is most prominent in fibers that express high levels of the catecholamine biosynthetic enzyme tyrosine hydroxylase (TH), whereas NET localizes to cytoplasmic compartments in axons relatively deficient in TH, suggesting a possible link of surface trafficking to NE synthesis capacity [36,49,50]. Interestingly, several investigators have provided evidence that NET traffics via dense core vesicles, the vesicles responsible for NE storage [49,51]. Thus, Kippenberger and colleagues [51] provided evidence that in PC12 cells NET-containing membranes co-fractionate with organelles competent for NE storage, presumably Golgi-derived large dense core vesicles [52]. Consistent with these findings, Schroeter and coworkers [49] provided evidence that the predominantly intracellular localization of NET in rat adrenal chromaffin cells involves localization to large dense core granules. Whereas Schroeter and coworkers also noted that NET is enriched at presynaptic sites colocalized with dopamine-beta hydroxylase, a protein component of dense core vesicles [49], these studies were not conducted at sufficient resolution to permit distinctions between subcellular compartments. Savchenko et al. demonstrated calcium-dependent increases in surface NET in neurons [53], strengthening the hypothesis that NET may be trafficked to the surface of NE neurons by fusion of dense core vesicles [53].

Like vesicular NE release, fusion of NET storage vesicles with the plasma membrane depends on SNARE machinery [43,54]. Thus, botulinum C1 toxin-mediated cleavage of syntaxin 1A reduces NET surface expression [43]. Moreover, NET grossly colocalizes with the SNARE protein syntaxin 1A in both CNS and sympathetic axons *in vivo *[43,49]. Possibly, NET stored on dense core vesicle membranes can traffic to the plasma membrane upon Ca^2+ ^influx, leading to incorporation of NET at release sites. However, Leitner and colleagues [48] provided evidence, using bovine splenic nerve preparations, that NET and the *bona fide *dense core vesicle marker, VMAT2, are localized to distinct populations of axonally transported vesicles. This study did not address or compare the organelles responsible for storing NET and NE at presynaptic terminals. Therefore, previous gross colocalization of NET and DBH at presynaptic compartments could be due to NET sorting to small dense core vesicles at these presynaptic terminals, since the biogenesis of small dense core vesicles occurs in the terminals [52,55,56].

To gain a better understanding of the subcellular distribution of NET, we developed a rodent-specific, NET monoclonal antibody (NET-05) and here demonstrate its suitability for the detection of native NET by both immunocytochemical and immunoblot techniques. We document NET-05 utility and specificity via staining of noradrenergic neurons in wildtype and NET knockout mice *in situ*. Further, we utilize this reagent in combination with other probes of NET and NET-associated proteins to gain insight into the subcellular distribution of NET within SCG axons and at the plasma membranes of SCG boutons. Our findings underscore a presynaptic enrichment of surface NET that colocalizes with syntaxin 1A and, in part, with lipid rafts. Importantly, using quantitative biochemical and immunocytochemical methods, we detect a clear segregation of NET from NE-containing dense core vesicles marked by VMAT2.

## Results

### Development and characterization of a rodent NET-specific monoclonal antibody

**Figure 1:**
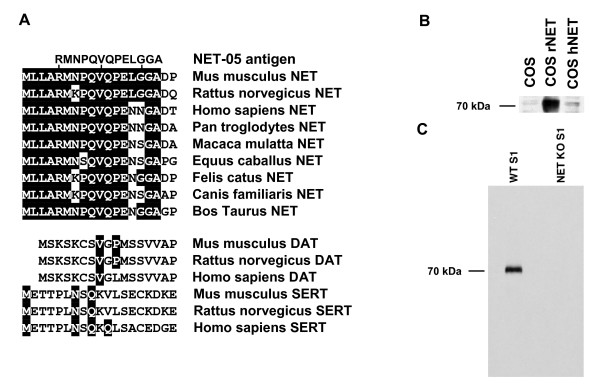
**NET-05 monoclonal antibody specifically recognizes rodent NET**. A. Alignment of the NET-05 peptide antigen with mNET amino terminus (starting at the first amino acid) and related monoamine transporters from various species. B. Representative Western blot analysis of cell extracts from nontransfected COS-7 cells or from COS-7 cells expressing either rat or human NET (rNET and hNET, respectively). C. Western blot assessment of NET expression in mouse brain (S1 fraction, cortex) using S1 membranes from either wild type or NET KO mice.

Given sensitivity and species detection issues inherent in our available NET-directed antibodies, we sought to generate a monoclonal antibody against the mouse NET (mNET) suitable for rodent-targeted Western blots, immunoprecipitation studies, and immunocytochemistry. We selected a sequence in mNET (amino acids 5–17) based on predicted antigenicity, and lack of conservation with other monoamine transporters such as the serotonin transporter (SERT) and dopamine transporter (DAT). This peptide is found in the N-terminal intracellular domain. Among NET paralogs, however, the mNET sequence chosen is highly conserved, differing from the rat NET (rNET) at only one amino acid (Figure 1A). Figure 1A provides an alignment of the NET amino termini from several species as well as the orthologs DAT and SERT from mouse, rat and human [57,58]. BLAST searches of mammalian protein databases yielded no significant matches to the epitope except those found in NET paralogs (data not shown). Following immunization with the coupled peptide (see Methods), we developed multiple hybridoma cell lines that produced antibodies yielding positive signals in peptide-based ELISA assays (data not shown). From these data, we selected a hybridoma (NET-05) for further characterization and use it throughout the experiments presented in this report.

Immunoblots using cell lysates from COS cells expressing rNET demonstrate that NET-05 recognizes rNET despite the one amino acid change relative to the mouse NET peptide (Figure 1B). However, NET-05 fails to detect human NET (hNET), which differs from mNET at two consecutive amino acids (Figure 1B). To verify whether NET-05 recognizes mNET in native tissue, we prepared post-nuclear fractions (S1) from wild type mouse cortex and from mice with mNET genetically deleted (NET KO). [28,59]. Our blots identify a ~70 kDa band present in wildtype but not NET KO samples (Figure 1C). These results demonstrate that NET-05 recognizes rodent NETs via Western blots of extracts from both heterologous expression systems and native tissues.

### Detection of specific NET expression in mouse brain noradrenergic neurons

**Figure 2:**
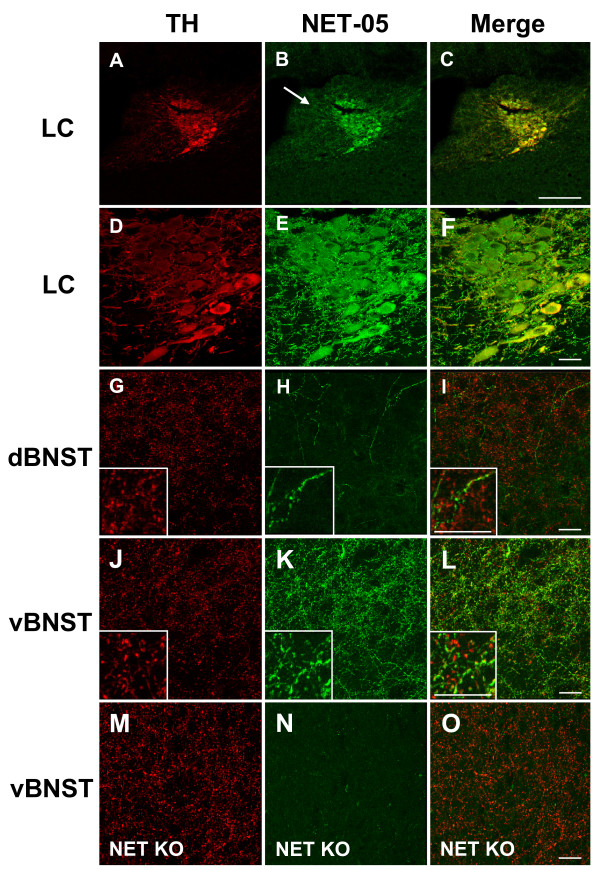
**NET colocalizes with TH in the locus ceruleus and bed nucleus of the stria terminalis**. Mouse brain sections were processed for confocal-assisted imaging of NET immunoreactivity as described in Methods. A-C. Coronal sections encompassing the locus ceruleus (LC) were doubled-labeled using antibodies against both tyrosine hydroxylase (TH, red) (A, D) and NET (B, E, green). Panels C and F are merged images of A/B and D/E, respectively. Arrow in B identifies a dual labeled LC dendrite. Scalebar in A-C = 200 μm; scalebar in D-F = 20 μm. G-I. Projections of six confocal sections (63×) taken from the dorsal BNST (dBNST). TH and NET immunoreactivities are shown in panel G (red) and panel H (green), respectively. Panel I is the merge of G and H. Insets of G-I show a digital zoom to illustrate individual fibers. J-L: Projections of six confocal sections (63×) taken from the ventral BNST (vBNST). TH immunoreactivity is shown in panel J (red) and NET immunoreactivity is shown in panel K and the overlap in panel L. All scalebar = 20 μM.

The distribution of noradrenergic neurons and processes in rodent brain is well-known [4] and the distribution of NET has been previously described in these pathways using polyclonal antisera [49,53,60]. To verify the utility of NET-05 for immunocytochemistry, we assessed the staining pattern obtained using NET-05, marking catecholamine neurons in parallel using anti-tyrosine hydroxylase (TH). As shown in a low magnification view (10×) of the locus ceruleus (LC), NET-05 immunoreactivity colocalizes extensively with staining for TH (Figure 2A–C) and extends into dendrites (arrow). Higher magnification (63×) views are shown in Figure 2D–F. NET-05 and TH staining similarly colocalize in the bed nucleus of the stria terminalis (BNST), an area rich in NE axons and varicosities (Figure 2G–L) [61-64]. NE fibers are more dense than in the ventral BNST (vBNST) as compared to the dorsal division (dBNST) [61-64]. Confirming this pattern, we observed a higher density of NET fiber labeling in the vBNST (Figure 2J–L) as compared to the dBNST (Figure 2G–I). Inset panels of Figure 2G–L using a stack from multiple confocal sections (n = 6) demonstrate NET labeling along individual fibers. Both TH and NET antigens appear enriched in punctate domains along individual fibers, presumably the well-known beaded varicosities that comprise NE release sites [4]. In these fields, some fibers are TH positive but show no NET immunoreactivity (see inset Figure 2I). These fibers are most likely dopaminergic fibers that express TH but lack NET and which are known to innervate this region [65]. To demonstrate specificity of the NET-05 reagent, we stained vBNST sections from wildtype and NET KO mice (Figure 2J–O). Although the pattern of TH staining in sections from NET KO (Figure 2M) appeared similar to that from wild type mice (Figure 2J), no NET staining was evident in the NET KO (Figure 2N). Similar negative staining results were obtained with sections incubated without primary antibody or pre-absorbed with peptide (data not shown).

### Subcellular distribution of neuronal NET in relation to VMAT2 in mouse brain

**Figure 3:**
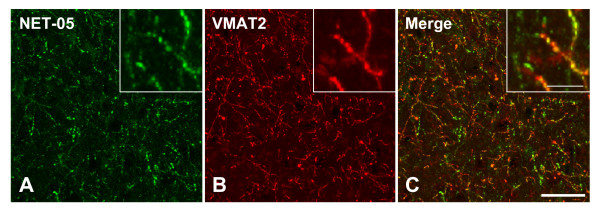
**NET immunoreactivity colocalizes with VMAT2 in the hippocampus**. Images were collected from the dentate gyrus of the mouse hippocampus. Sections were immunostained using NET-05 (A, green) and VMAT2 antibody (B, red). Panel C is a merge of A and B. Extensive colocalization of VMAT2 and NET is evident on many fibers. Essentially all VMAT2 positive puncta colocalize with NET immunoreactivity. In contrast, some NET-positive fiber segments appear to have low or absent VMAT2 staining (note green profiles in C inset). Insets shows a digital zoom of each field. Scalebar A-C = 50 μM. Scalebar for inset panels = 10 μM.

Having validated the utility of NET-05 antibody for detection of mouse brain NET in multiple assays, we examined the hypothesis that NET is sorted to dense core vesicles that harbor VMAT2. Figure 3 illustrates the distribution of NET (Figure 3A) and VMAT2 (Figure 3B) immunoreactivity in the mouse hippocampus, focusing on the NE rich hilus of the dentate gyrus. Labeling of NET in these fields is similar to that achieved with polyclonal NET antisera [49,53]. Although colabeling was evident in most neuronal varicosities, colocalization was not uniform as some NET-positive elements displayed low levels of VMAT2 immunoreactivity and some varicosities appeared to have high levels of VMAT2 relative to NET. Regardless, staining in the hippocampus, as in the LC and BNST, is absent in the NET KO (data not shown), confirming NET-05 specificity. These light-level immunocytochemical data confirm expression of NET at discontinuous axonal in the CNS and indicates that the relative abundance of NET and VMAT2 differs across individual fibers.

### NET distribution in cultured superior cervical ganglion (SCG) neurons

**Figure 4:**
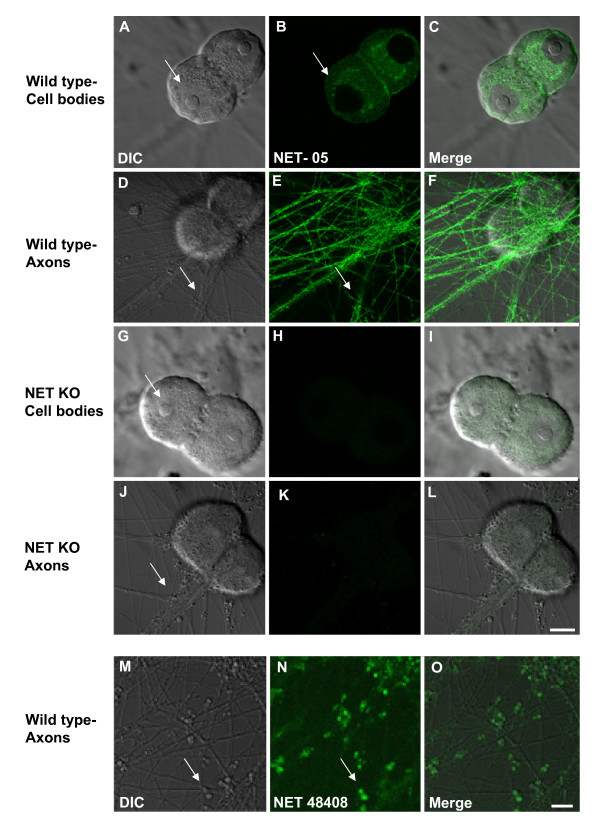
**Visualization of NET expression in cultured SCGs**. Cultured mouse SCGs were immunolabeled with monoclonal NET-05 (permeabilized) or NET 43408 (nonpermeabilized) antibodies. Shown are single confocal sections and corresponding DIC images from SCG cultures stained with NET-05 antibody and obtained from WT (A-I) or NET KO (J-L) animals. NET-05 recognizes NET in both cell bodies (B) and in axons (E), as noted by arrows. No signal was detected in either cell bodies or axons in the NET KO neurons (H, K). Scale bar = 10 μM. M-O: Staining of SCG cultures under nonpermeabilized conditions with ectodomain NET antibody 43408. Note selective localization of NET staining to the surface of SCG boutons (arrows). Scale bar = 10 μM.

Although we achieved evidence of specific NET labeling within or on neuronal membranes, the small size of CNS NE varicosities, unfortunately, precluded quantitative immunocytochemical analysis of NET distribution. Previously, we observed that NET is enriched in sympathetic varicosities *in situ *[43,53]. Therefore, we continued our studies using SCG neurons that elaborate profuse noradrenergic fibers in culture [49,53]. Furthermore, SCG preparations present large varicosities extending laterally from axonal membranes (for example see 8A). As with the CNS preparations described above, we verified the specificity of NET-05 in the SCG cultures and then examined NET subcellular distribution in relation to multiple markers of vesicles and membrane subdomains. Figure 4 shows single confocal sections and corresponding DIC images of cultured SCGs focused on either the cell bodies (Figure 4A–C) or the processes (Figure 4D–F). In the cell bodies, NET labeling surrounds the nucleus in a pattern consistent with Golgi compartments. A low level of immunoreactivity was associated with the plasma membrane, most evident at sites where cell bodies contact one another (Figure 4B). Importantly, NET-05 labeling exhibited a punctate pattern throughout SCG processes (Figure 4E). To demonstrate specificity of NET-05 labeling in this preparation, we stained SCG cultures prepared from NET KO mice (Figure 4G–L). As shown in Figure 4H and 4K, the KO neurons lack NET staining.

Next, we compared the distribution of NET as detected with NET-05, which requires permeabilization, to that of NET 43408, a polyclonal NET antibody that targets the NET 2^nd ^extracellular loop and which therefore reports NET expression at the cell surface when used in the absence of detergent [53]. Whereas NET-05 detects transporter throughout neuronal processes (Figure 4E), NET 43408 reveals NET surface expression particularly evident at boutons (Figure 4N, arrows, for higher magnification, see Figure 8a–d). These boutons store NPY (see below) and thus may represent one class of NE neurons that innervate smooth muscle [66,67].

### SCG NET does not colocalize with markers of dense core vesicles (DCVs)

**Figure 5:**
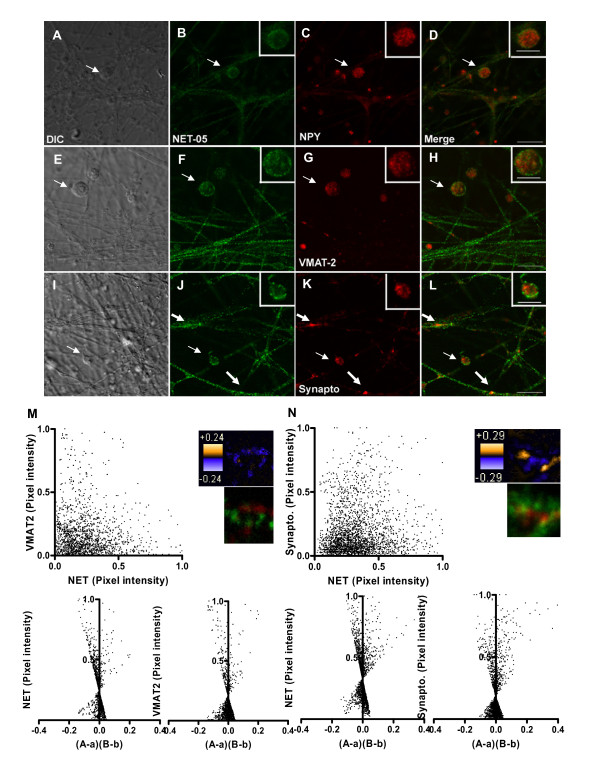
**NET labeling segregates from markers of large and small dense core vesicles**. SCGs were double-labeled with NET-05 and antibodies directed against VMAT2, NPY or synaptophysin. The first column (A, E, and I) represents the DIC images of an exemplary field of cells, whereas the second column (B, F, J, green) is the distribution of NET in the same or fibers or boutons. The fourth column (C, G, K, red) are images obtained with antibodies against NPY, VMAT2, or synaptophysin (Synapto). The last column (D, H, and L) represents the overlap of the signals obtained with NET-05 and the particular granule protein targeted. Scale bar = 10 micron. Insets in staining panels provide higher power views of single boutons. Scale bar = 5 micron. M. Correlation of NET and VMAT2 pixel densities (upper left), ICA analysis (lower panels), and spatial display of PDM values (upper right). N. Correlation of NET and synaptophysin pixel densities (upper left), ICA analysis (lower panels), and spatial display of PDM values (upper right). PDM color transformation is shown above standard red/green visualizations of same field.

The studies above indicate that the NET-05 labeling associated with SCG neurites, in the presence of detergent, represents largely intracellular pools of transporter-containing membranes, whereas boutons are the primary sites of surface NET expression. To test if intracellular NET resides on large dense core vesicles, we double stained SCGs for NET and NPY (a neuropeptide sorted to large dense core vesicles in SCGs [66,68]. As indicated above, NET-05 labeled the surface and intracellular membranes of single SCG boutons (Figure 5A, B). NPY labeling was enriched in the cytoplasm of boutons and relatively low in abundance within processes, relative to that observed for NET (Figure 5C). Importantly, we observed no colocalization with NET in boutons (Figure 5A–D see arrows and Figure 5D inset) suggesting NET is not sorted to large dense core vesicles (LDCVs). We considered the possibility that bouton resident NET vesicles might represent small dense core vesicles (SDCVs) which originate at presynaptic sites. Upon fusion with the plasma membrane, LDCVs release NPY and LDCV membrane proteins, including VMAT2, are endocytosed to form SDCVs [52,55,56,69]. Just as we observed for NPY, VMAT2 labeling was also enriched in the cytoplasm of SCG boutons and in relatively low abundance in processes as compared to NET (Figure 5G). Importantly, within the bouton of double labeled neurons, VMAT2 displayed little apparent colocalization with NET (see arrows and Figure 5H inset). Additionally, we found little if any colocalization of synaptophysin, which labels an endocytic compartment through which SDCVs [52,56,70] (Figure 5I–L, 5L inset). Together these findings support the contention that in SCG boutons, NET is not sorted to any class of dense core vesicles.

**Figure 6:**
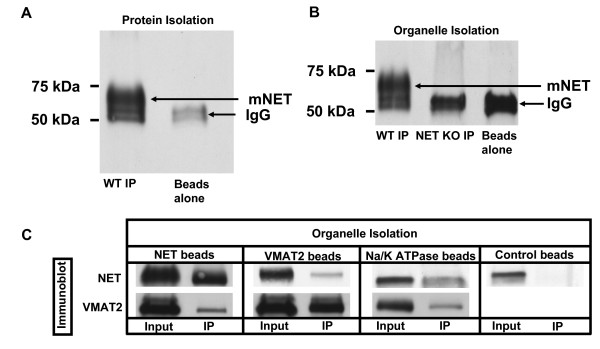
**Segregation of NET and VMAT2 detected via immunoisolation of SCG vesicles**. Western blot analysis of immunoisolated membranes captured with NET, VMAT2 or Na/K ATPase antibodies. A. Western blot analysis of NET-05 immunoprecipitates from solubilized mouse cortical synaptosomes (solubilized P2 fraction probed with NET-05 to reveal NET). B. NET immunoreactivity associated with vesicles obtained from lysed mouse cortical synaptosomes from wildtype or NET KO mice, immunoprecipitated with paramagnetic anti IgG beads coated with NET-05 antibody, or beads alone. Panel C shows Western blot analysis of immunoprecipitations of organelles from SCG neuron by beads coated with NET antibodies (organelle isolation, NET beads) and Na/K ATPase antibodies (organelle isolation, Na/K ATPase beads) or VMAT2 antibodies (organelle isolation, control or VMAT2 beads). Elutants of immunoprecpitations are probed with antibodies against either NET or VMAT2.

The evaluations presented above of NET colocalization with VMAT2 and other proteins are qualitative, based on visual inspection of chromatic changes in labeling when using multiple fluorophore-labeled secondary antibodies. To investigate colocalization quantitatively, we plotted the normalized intensities for NET and VMAT2 (Figure 5M upper left plot) or NET and synaptophysin (Figure 5N upper left plot) in both axonal regions lacking varicosities as well as in boutons, and found no evidence of correlation. To achieve a quantitative evaluation of colocalization, we implemented an unbiased analytical method that has been validated for determination of protein colocalization in intact cells [71]. Stanley's group reasoned that if two proteins are on the same organelle or interacted, then the paired pixel intensities should vary in synchrony over space [71]. This method, termed Intensity Correlation Analysis (ICA) plots the normalized pixel intensity for a particular protein (e.g. NET) in relation to the normalized intensity of the other protein (e.g. VMAT2). The quantitative index produced in the ICA analysis is termed the Product of the Difference from the Mean, PDM [71]. In graphical form, the normalized paired pixel intensity of each protein (e.g. NET) is plotted against the PDM of the two proteins of interest, producing a distribution of points between the values of 0 and 1 on the y axis and between -0.5 to +0.5 on the x axis (e.g. Figure 6M and 6N bottom panels). Pixel intensities that indicate colocalization are associated with positive values of the x axis. For statistical analysis, the number of pixels with a positive × value is divided by the total number pixels. This procedure yields the intensity correlation quotient (ICQ) which can be analyzed by a nonparametric sign test [71]. Analysis of NET and VMAT2 yielded a nonsignificant ICQ value (P > .05) consistent with NET and VMAT2 as localized to different membrane compartments. The ICQ value for NET and synaptophysin in axons is low, though a significant association was identified (P < 0.001) (see arrow in Figure 5L).

In Figure 5M and 5N, we present the spatial distribution of PDM values (upper right panels) using a pseudocolored scale. Pixels with positive PDM values are in orange (high colocalization) and negative PDM values in purple (segregation). Below this display, we present the standard two-color merge of the same region of the axon. Whereas NET and VMAT2 show no extensive regions of colocalization, for NET and synaptophysin, limited regions within the axon appear to demonstrate positive colocalization (Figure 5, thick arrows). However, when we performed these analyses in boutons, we detect no evidence of NET colocalization with VMAT2, NPY, or synaptophysin (data not shown), further confirming segregation of NET from LDCVs in axons as well as SDCVs in boutons.

### Immunoisolation studies support NET and VMAT2 localization to distinct SCG vesicles

To evaluate the distribution of NET and VMAT2 via an alternative approach, we performed immunoisolation experiments of SCG membranes. To establish the utility of NET-05 for immunoprecipitations, we incubated antibody with detergent extracts of cortical synaptosomes and probed NET with the same antibody (Figure 6A). Immunopreciptated NET migrates at ~70 kDa in total extracts (Figure 1), migrating on SDS-PAGE just above the heavy chain of NET-05 IgG (52 kDA) (see Figure 6A; beads alone). Additionally, we also immunoprecipitated intact organelles from hypotonic lysates of mouse cortical synaptosomes (prepared in the absence of detergent). The 70 kDa NET band observed in wildtype (WT) but not NET KO lysates demonstrates specific NET recovery (Figure 6B). In Figure 6C, we present results of organelle isolation from cultured SCGs using NET-05, VMAT2 or Na/K ATPase antibodies for membrane isolation, followed by blots of NET and VMAT2. NET-05 isolated membranes contained only background levels of VMAT2 (comparable to that obtained with Na/K ATPase coated beads). Conversely, VMAT2-coated beads recovered negligible, background levels of NET proteins. Antibody-free beads yielded negligible background NET isolation. These findings support the contention derived from staining experiments in SCG cultures that NET and VMAT2 are segregated to distinct membrane compartments.

### Colocalization of NET with SCAMP2 and syntaxin 1A in SCG axons

**Figure 7:**
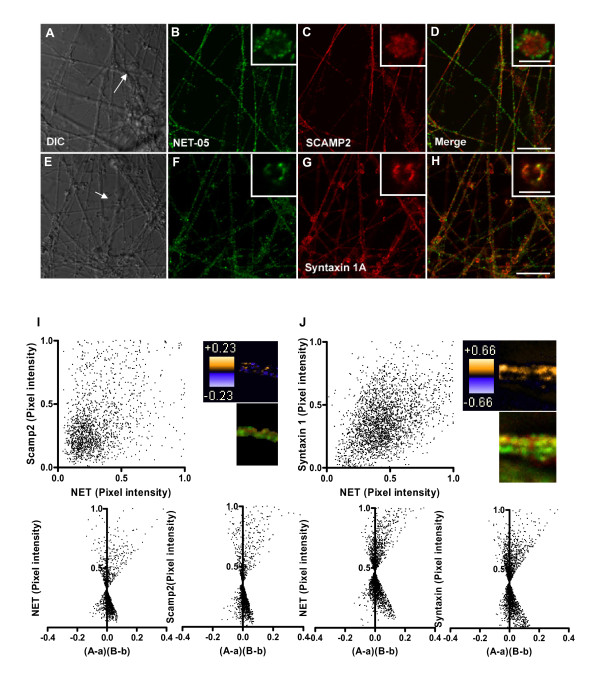
**NET colocalizes extensively with syntaxin 1A but less so with SCAMP2**. SCGs were double-labeled with NET-05 and with antibodies directed against syntaxin 1A or SCAMP2. The first column (A, and E) represents the DIC images of an exemplary field of cells whereas the second column (B, F, green) is the distribution of the NET in the same or fibers or boutons. Arrow in A highlights bouton shown in inset. The third column (C, G, red) shows images obtained with antibodies against syntaxin 1A or SCAMP2. The last column (D, H) represents the merge of signals obtained with NET-05 and the appropriate synaptic membrane proteins. Insets in staining panels provide a higher power view of single boutons and are highlighted in full images with arrows. I. Correlation of NET and SCAMP2 pixel densities (I, upper left), ICA analysis (lower panels), and spatial display of PDM values (I, upper right). J. Correlation of NET and syntaxin 1A pixel densities (upper left), ICA analysis (lower panels) and spatial display of PDM values (J, upper right). PDM color transformation is shown above standard red/green visualizations of same field.

Recently, SCAMP2 has been identified as an N-terminal SERT and DAT interacting protein, with suggestions of a role in transporter sorting or trafficking [72]. It seemed likely that SCAMP2 might also associate with NET as well, and thus we explored NET and SCAMP2 colocalization in SCG cultures (Figure 7A–D). SCAMP2 immunoreactivity was readily detected in both axons and boutons. In boutons, we observed no statistically significant colocalization with NET (Figure 7A–D). Likewise, neither the PDM image of a representative axon nor the two color merge of the same segment (Figure 7I, top right) gives evidence of strong colocalization between NET and SCAMP2. The ICA plot (Figure 7I bottom) appears symmetric with a "cloud" of points in the lower quadrant. Nonetheless, the ICQ value (+0.027) reflects a significant association (P = 0.01) that supports the possibility that a small fraction of NET resides on SCAMP2-positive organelles or that a minor fraction of SCAMP2 resides on NET-transport vesicles.

We have previously demonstrated that NET colocalizes with syntaxin 1A in sympathetic varicosities *in situ *[43,49,53]. Indeed, we gained clear evidence in the SCG cultures for extensive colocalization of syntaxin 1A and NET (Figure 7E–H) at the surface of boutons. Since it is unknown whether NET and syntaxin 1A are sorted to distinct axonal transport vesicles, we examined the correlation of these two proteins in axons. Visually, we observed apparent colocalization of NET and syntaxin 1A. Indeed, the distribution of pixel intensities for axonal NET and syntaxin 1A (Figure 7J top) demonstrates a positive spatial correlation for this labeling. ICA/ICQ analysis confirms a significant association of axonal NET and syntaxin 1A (P ≤ 0.001). When a similar analysis was performed in boutons, again the two proteins exhibited a highly statistically significant colocalization (data not shown), consistent with previous biochemical studies [43]. These findings indicate that NET is sorted to syntaxin 1A containing vesicles such that both SNARE protein and transporter can be deposited at the plasma membrane upon membrane fusion.

### NET localizes to both lipid raft and non-lipid raft membranes in SCGs

**Figure 8:**
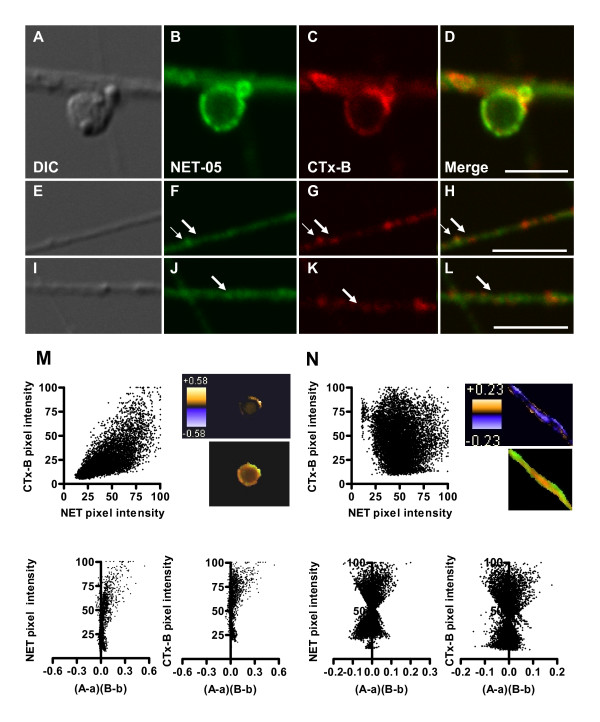
**NET localizes to lipid rafts at the bouton surface but not to raft domains labeled within axonal membranes**. SCGs were double-labeled with NET-05 and with fluorescent cholera toxin B (CTx-B). SCG's were either labeled with CTx-B prior to (A-D), or after detergent permeabilization (E-H), followed by NET-05 labeling. Small arrows indicate colocalization whereas large arrows point to regions of segregation. Correlation of NET and CTx-B pixel intensities using nonpermeabilized conditions (M, upper left), ICA analysis (lower panels) and spatial display of PDM values (M, upper right). N. Correlation of NET and CTx-B pixel intensities using permeabilizing conditions (upper left), ICA analysis (lower panels) and spatial display of PDM values (N, upper right). Scale bar = 5 μM. PDM color transformation is shown above standard red/green visualizations of same field.

Plasma membranes are not homogenous lipid bilayers but rather contain discrete subdomains rich in cholesterol and sphingolipids termed lipid rafts [73,74]. Interestingly, biochemical fractionation and pharmacological studies of placental trophoblasts demonstrate that a portion of NETs exists in lipid rafts [46]. Whether neuronal NET localizes to these domains has not been established. Cholera toxin-B (CTx-B) targets cell-surface localized GM1 gangliosides that are enriched in lipid-rafts [75]. We used fluorescent CTx-B to label either nonpermeabilized or detergent-permeabilized SCGs to determine if NET localizes to lipid raft compartments. As shown in Figure 8A–D, fluorescent CTx-B labeling of SCG processes and boutons under nonpermeabilized conditions was nonuniform as expected of lipid raft membrane subdomains. Remarkably, all sites labeled with CTx-B under nonpermeabilized conditions displayed NET05 immunoreactivity (Figure 8A–D). However, the converse was not true, demonstrating that NET in the plasma membrane of boutons localizes to both raft and non-raft membranes. In permeabilized conditions, CTx-B labeling of processes revealed little colocalization with NET (Figure 8E–L, see thicker arrows). Consistent with these observations, the spatial distribution of pixel intensities for NET and CTx-B (Figure 8M top) demonstrated a positive correlation for nonpermeabilized boutons whereas axonal labeling under permeabilized conditions exhibited a random pattern is observed (Figure 8N top). ICA/ICQ analysis yielded a significant association of NET and CTx-B in nonpermeabilized (ICQ = 0.315; P < 0.001) as compared to permeabilized axonal preparations (ICQ = -0.032; P > 0.05) (Figure 8M and 8N). These findings support the targeting of NET proteins to lipid rafts once transporters have been inserted into the presynaptic membrane.

## Discussion

In this report, advance the understanding of NET subcellular distribution in neuronal membranes. We desired to combine immunohistochemical and biochemical strategies, and thus needed first to develop and characterize a high-affinity, rodent NET-directed antibody. Our two previous NET rabbit polyclonal antibodies, NET43411 [49] and NET 43408 [53] detect rodent NET in intact tissue, but do not reliably detect NET by Western blotting nor allow for NET immunoprecipitation. Previously, we developed an anti-human NET monoclonal antibody that has the required sensitivity for blotting studies, but fails to detect rodent NET due lack of epitope conservation across species. Here, we demonstrate that the NET-05 monoclonal antibody provides for immunoblotting, immunoprecipitation and immunocytochemical localization of mouse NET with sufficient sensitivity to detect NET in native preparations. Lack of signal in assays using tissues obtained from NET KO mice amply demonstrates the specificity of the NET-05 reagent.

As expected, NET-05 stains well-known areas of noradrenergic cell bodies and projections, including the LC, BNST, and hippocampus, where fiber staining for both VMAT2 and NET exhibits a characteristic varicose appearance. Interestingly, some NET-positive fibers appeared to lack, or contained little VMAT2, possibly an indication of low capacities of these fibers for vesicular NE release. EM-immunocytochemical studies [36,50] in the cortex have shown that NET expression patterns exhibit one of two patterns, being either largely surface localized or predominantly intracellular. Surface localized NET is found in fibers with high levels of TH whereas intracellular NET predominates in fibers with little or no detectible TH immunoreactivity. Possibly the VMAT2-poor, NET labeled fibers we observe in the hippocampus also reflect fibers with reduced NE release capacity. Further studies utilizing EM approaches and dual labeling for NET/VMAT2 should be helpful in addressing this possibility as well as advancing the possibility that such a pattern may change with stress or other states of behavioral activation [36].

Because CNS NE fibers and their varicosities are too small for detailed compartmental analyses, we turned to SCG cultures. In SCG cell bodies, NET labeling displays a pattern consistent with ER/Golgi localization as well as a low amount of surface expression at regions where cell bodies appear to contact with each other. As previously shown[53]., NET ectodomain antibodies reveal surface expression at varicosities but not on axonal plasma membranes. In contrast, when we labeled NET-05 under permeabilizing conditions, we observed uniformly intense staining of NET throughout axons. These data suggest that NET-05 labeling in SCG axons primarily reveals the presence of intracellular NET transport vesicles. Interestingly, the intensity of intracellular staining was much lower in single SCG boutons, whose diameter (~5 μm) permitted an unprecedented level of analysis of NET distribution in relation to other presynaptic membrane proteins. An additional benefit of studying NET distribution in these large boutons is that they project laterally from the axons, permitting visualization of both axonal and presynaptic organelles in the same field.

To study NET colocalization with synaptic membrane proteins, we took advantage of a recently developed method for determining colocalization of proteins in intact cells (ICA/ICQ; [71]. This analysis allows for the characterization of the subcellular domain in which two proteins either physically interact or are colocalized to a common organelle or membrane domain. As ICA/ICQ analysis utilizes an optical approach with non-extracted membranes, this approach provides an important complement to biochemical analyses that utilize solubilized membranes and denatured preparations.

One of the aims of our study was to determine if NET is sorted to dense core vesicles. Dense core vesicles can be placed into two classes based on site of biogenesis and size. Secretory vesicles in NE neurons bud from Golgi membranes in the cell soma and identified as LDCVs. These vesicles contain VMAT2 to provide for DA import prior to NE synthesis by intragranular DBH. Many NE neurons, including SCGs, also sort neuropeptides to LDCVs [52,68]. At synaptic sites, LDCVs fuse with the plasma membrane, where vesicular membrane constituents can recycle via smaller, endocytic compartments that contain synaptophysin. These SDCVs lack neuropeptides but retain VMAT2 and DBH [52]. We found no evidence of colocalization of SCG NET with NPY, a neuropeptide sorted to LDCVs, clearly demonstrating early segregation of NET from secretory granule membrane components. We also found that NET also does not colocalize with axonal VMAT2. Our findings agree with a prior study that utilized density-based fractionation techniques to examine codistribution of NET and VMAT2 in axons [48]. Our studies utilized an intact preparation with both qualitative and quantitative inspection of NET and VMAT2 in axons and boutons. Additionally, using immunoisolation of intact organelles, we found no evidence of extensive colocalization of NET and VMAT2. Our studies indicate that in SCG neurons, NET sorts to trafficking vesicles distinct from either LDCVs or SDCVs. Despite this early segregation, both organelles target to presynaptic boutons and can fuse with the plasma membrane [53].

An attractive organelle to support NET trafficking is synaptic-like microvesicles (SLMVs). These synaptophysin-rich vesicles can fuse in a calcium-dependent manner and in some NE cells can also store and release acetylcholine [56,76,77]. However, we did not detect any colocalization of NET with synaptophysin at boutons and we have not detected any colocalization of NET with the presynaptic choline transporter that is present in these terminals and presumably is sorted to SLMVs (data not shown). Nonetheless, ICA/ICQ analysis of permeabilized SCG axons showed that a small but significant fraction of synaptophysin containing membrane colocalizes with NET. Interestingly, these synaptophysin/NET containing organelles do not appear to concentrate at boutons (Figure 7) and thus could represent constitutive trafficking organelles that deliver NET to boutons. Indeed, synaptophysin has been suggested to traffic via constitutive transport organelles [52,70,78,79]. Regardless, these synaptophysin- and NET-containing vesicles do not appear to lead to the sorting of NET to SDCVs or SLMVs following fusion as we did not observe any colocalization of NET with synaptophysin inside boutons. Possibly, NET-and synaptophysin-positive membranes could also represent retrograde compartments formed from the fusion of separate NET and synaptophysin-containing endosomes that are destined for degradation. Further biochemical studies of surface-labeled NET vesicles should help address this issue.

An additional issue underlying our studies is whether proteins that associate with NET do so early in their trafficking itinerary or only after plasma membrane insertion. A contribution to this question can be seen in our analysis of the distribution of NET to syntaxin 1A with NET. Like NET, syntaxin 1A has uniformly high levels in permeabilized axons relative to the boutons. Indeed, in permeabilized preparations, we observed significant and extensive colocalization of NET with syntaxin 1A. From previous work, it is known that syntaxin 1A is required for basal/regulated NET cell surface expression and that NET and syntaxin 1A physically associate [35,43,54]. Our findings are consistent with the existence of trafficking vesicles that contain both NET and syntaxin 1A, providing an opportunity for physical interactions that ultimately serve to regulate NET activity and channel states at the plasma membrane. Recent studies with a *C. elegans *NET homolog, (DAT-1), reveals that a transporter N-terminal GFP tag can disrupt syntaxin 1A interaction and lead to altered channel states and membrane potential, providing evidence for a physiological relevance of the syntaxin 1A/transporter interaction *in vivo *[80].

Finally, we examined the distribution of NET in relation to two markers of membrane subcompartments that could support transporter NET trafficking and/or regulation, SCAMP2 and CTx-B. A previous study reported that SCAMP2 physically interacts with both SERT and DAT. SERT and SCAMP2 colocalize in intracellular compartments, as well as at the plasma membrane where it may regulate SERT activity [72]. We found SCAMP2 to be highly expressed in SCG processes where we detected a very low, but significant, level of NET colocalization suggesting that SCAMP2 may participate in sorting and export of the transporter from the cell soma. Like DAT, NET lacks a PY motif [81,82] and thus NET/SCAMP2 associations at the plasma membrane could facilitate NEDD4 ubiquitination, similar to the role suggested for SCAMP3 in NEDD4 ubiquitination of EGF receptors [83]. We detected, membrane patches at the surface of boutons that were enriched for both NET and SCAMP2. Compared to the total level of SCAMP2 in boutons, this distribution was quite limited. Additional studies that explore conditions leading to NET ubiquitination should be helpful in further evaluating the extent and role of NET/SCAMP2 associations. Placental cells that express NET localize a portion of NET to lipid rafts as detected through membrane fractionation analyses [46,47]. In SCG neurites examined under permeabilized conditions, NET does not colocalize with CTx-B. In boutons, in contrast, the transporter clearly localizes to surface-labeled, CTx-B positive membrane domains. As a portion of NET in CTx-B unlabeled membranes, we suggest that NET may transit between these domains as part of a regulatory cycle. Future studies that explore the dynamics of these associations and that take advantage of the reagents and paradigms employed in our study should further extend our understanding of the regulation of NET membrane trafficking.

## Conclusion

Our findings support the hypothesis that SCG NET is segregated prior to transport from the cell body from proteins comprising large dense core vesicles. Once localized to presynaptic boutons, NET does not recycle via VMAT2-positive, small dense core vesicles. Finally, once NET reaches presynaptic plasma membranes, the transporter localizes to syntaxin 1A-rich plasma membrane domains, with a portion found in cholera toxin-demarcated lipid rafts. Our findings indicate that activity-dependent insertion of NET into the SCG plasma membrane derives from vesicles distinct from those that deliver NE. Moreover, NET is localized in presynaptic membranes in a manner that can take advantage of regulatory processes targeting lipid raft subdomains.

## Methods

### Antibody development

We and others have developed polyclonal antisera capable of detecting NET protein by immunocytochemical techniques *in situ *[49,53,60,84,85]. Prior to this study, we had also developed a high-affinity, mouse monoclonal antibody that targets human NET (hNET) [86]. A monoclonal antibody suitable for colocalization studies with other polyclonal reagents in rodent preparations and sensitive enough for detection of native protein by immunoblots was not available, prompting our current efforts. We thus generated the mouse NET amino terminal peptide RMNPQVQPELGGA (amino acids 5–17) via solid-phase techniques (Tufts University Core Facility), adding a C-terminal cysteine to facilitate coupling to keyhole limpet hemocyanin (KLH). Subsequent efforts using animals for antibody development and characterization were performed with attention to the *NIH Guide for the Care and Use of Laboratory Animals *under protocols approved by Institutional Animal Care and Use Committees of the Vanderbilt University School of Medicine. Mouse monoclonal antibodies to the NET peptide conjugate were prepared following standard hybridoma techniques [87] in collaboration with MAb Technologies (Mabtechnologies.com). Briefly, 3 BALB/c mice were given subcutaneous and intraperitoneal primary injections with the KLH conjugate emulsified in Freund's complete adjuvant. Subsequent booster shots were given subcutaneously at 3–4 week intervals until a strong immune response to the antigen was produced, as assessed via ELISA assays on tail vein blood. The best responder was sacrificed, the spleen was isolated and spleen cells were extracted and fused to a mouse myeloma cell line (P3X63.Ag8.653) using polyethylene glycol (PEG). Cells were plated in 8 × 96 well plates under hypoxanthine-aminopterin-thymidine (HAT) selection, supplementing with peritoneal macrophage feeder cells. We screened the culture medium from individual wells for the presence of antigen secreting cells, as assessed by ELISA assays as well as Western blots of mouse and rat NET transfected cells. Cells from wells that tested positive were cloned by limiting dilution in 96 well plates. Hybridomas in positive wells from the cloning plates were again re-cloned by limiting dilution to establish a final sub-cloned antibody producing hybridoma cell line (NET-05). Isotype analysis revealed the immunoglobulin secreted to be of the IgG_2b _subtype.

### Additional antibodies

To evaluate of the sensitivity and specificity of NET-05, we compared results to our hNET-specific monoclonal antibody (NET17-1; MAb Technologies, 1:1000) [86,88] as well as a rabbit anti-NET polyclonal antiserum, designated 43408 [53]. The antigen for NET 43408 is a KLH conjugated peptide derived from the transporter's 2^nd ^extracellular loop. This anti-NET polyclonal antiserum (43408) labels mouse noradrenergic neurons in the CNS and periphery that is lost with preparations from NET KO mice [53]. Additional commercially available reagents used included anti-tyrosine hydroxylase (TH, Chemicon, 1:3000); anti-NPY (Santa Cruz 1:200), anti-vesicular monoamine transporter 2 (VMAT2 Chemicon and Santa Cruz 1:1000), anti-synaptophysin (synapto, Boehringer Mannheim, 1:300) and anti-dopamine beta hydoxylase (DBH, Chemicon 1:300). Anti-SCAMP2 was gift of Dr David Castle (University of Virginia). Secondary antibody reagents included donkey anti-rabbit IgG conjugated with Cy3 or Cy2 (Jackson Labs, 1:1000), donkey anti-mouse IgG conjugated with Cy2 (Jackson Labs, 1:500), goat anti-rabbit conjugated with Alexa 568 (Molecular Probes, 1:1000), goat anti-mouse conjugated with Alexa 488 or Alexa 568 (Molecular Probes, 1:1000), goat anti-rmouse IgG2_b _conjugated with Alexa 488 (Molecular Probes, 1:1000) and goat anti-rmouse IgG1 conjugated with Alexa 568 (Molecular Probes, 1:1000). Alexa Flour 488 conjugated cholera toxin subunit B was from Molecular Probes.

### Western and immunoprecipitation analysis

Western analysis and immunopreciptations were performed as previously described [35,43,54]. Briefly, cortical synaptosomes were prepared [89,90] and either lysed in 1% Triton X-100 for one hr or lysed in hypotonic buffer [89,91]. Membranes from cultured superior cervical ganglion neurons (SCGs) were also prepared by hypotonic lysis to release synaptic vesicles and other cytoplasmic organelles as in Nagy et al and modified by Huttner and coworkers [89,91]. This method involves a step in which membranes are incubated with buffered water allowing release of intact synaptic vesicles [89,91]; however, larger organelles may be lysed. Detergent extracted membranes (1 mL) were cleared of non-solubilized membranes by a 30 minute centrifugation at 100,000 × *g *and 10 uL NET-05 was added. After a 2 hr incubation, extracts were incubated with Protein A beads (Sigma) for 1 hr. Beads were washed and eluted with Laemmli buffer [92]. Synaptosomes (1 mL) or SCGs lysed with hypotonic media were used after the addition of 150 mM NaCl and centrifugation at 1000 × *g *for 20 minutes [89]. Membranes were incubated for 4 hrs with 10 uL NET-05 and then incubated for 2 hr with anti-mouse paramagnetic beads (Dynal; Invitrogen). Beads were washed in buffer 5× and then eluted with Laemmli buffer prior to separation of proteins by SDS-PAGE and transfer to Polyvinylidene Difluoride (Biorad, PVDF 0.2 micron) for immunoblotting. NET was detected using NET-05 and immunoreactivity was observed using chemiluminescence (Western Lightning Plus-ECL, PerkinElmer) insuring exposures were conducted in the linear range of exposed X-ray film.

### Detection of NET *in situ *by immunofluorescence

Adult (8–12 weeks) wild type male mice (C57Bl/6J) and age-matched NET KO mice [28,59] were used to examine CNS NET distribution whereas P0–P5 pups where used for the culturing of neurons from the superior cervical ganglion as outlined below. NET KO mice were generously provided by Dr. Marc Caron (HHMI, Duke University School of Medicine). Animals were housed in 14-h/10-h light/dark cycles with food and water *ad libitum*. Mice were deeply anesthetized with Nembutal (80 mg/kg), and transcardially perfused with saline containing 4% paraformaldehyde. Brains were removed and immersed in fixative for two to forty eight hours at 4°C and then cryoprotected in 30% sucrose overnight at 4°C. Thirty or forty-micrometer-thick floating frozen sections were cut into PBS using a sledge microtome (Leica) and then incubated sequentially with primary antibodies (room temperature, 4 hr or forty eight hours at 4°C) and secondary antibodies (room temperature, 2 hr or twenty four hours at 4°C). Sections were permeabilized with phosphate-buffered saline (PBS) containing 0.15% or 0.2% TX-100. Nonspecific labeling was blocked by incubation in PBS containing 4% bovine serum albumin and 0.15% NP-40 or PBS containing 4% donkey serum for 1 hr prior to incubations with antibody solutions containing 4% bovine serum albumin and 0.05% NP-40 or 0.1% TX-100 and 2% donkey serum. Controls included incubations in the absence of primary and secondary antibody reagents and as well as the use of sections prepared from the brains of NET KO mice. Immunofluorescence was visualized using a Zeiss LSM 510 Meta confocal imaging system equipped with internal He/Ne and external Ar/Kr lasers (VUMC Cell Imaging Core Resource, Sam Wells Director, supported by NIH Grants CA68485 and DK20593) with output at 488 (Cy2, Alexa 488), 568 (Cy3, Alexa 568) and 633 (Cy5, Alexa 633) nm. *Z*-series were collected by optical sectioning at intervals ranging from 0.25 to 1 μm depending on the magnification used, followed in some cases by 3D reconstruction. Image processing and montage assembly were performed using either Zeiss or Metamorph software and Adobe Photoshop. Designation of anatomical structures for dissection and immunostaining followed regional and nuclear designations of [93].

### Culture of mouse superior cervical ganglion neurons (SCGs)

SCGs were cultured as previously described [43,53]. Briefly, ganglia from 0–5 day old wild type pups (C57Bl/6J) and NET KO pups were dissected and incubated for 30 min at 37°C in collagenase (3 mg/ml; Sigma) and trypsin (0.5 mg/mL/Gibco) followed by termination of digestion using 10% fetal bovine serum (FBS, Atlanta Biologicals) in UltraCulture medium (BioWhittaker). To purify SCGs cells away from fibroblasts, cells were incubated in UltraCulture medium supplemented with 3% FBS, nerve growth factor (NGF, 20 ng/mL; Harlan) and 2 mM L-glutamine for 2 h at 37°C, SCGs were removed, centrifuged for 5 min, resuspended in supplemented UltraCulture medium and then transferred onto poly-D-lysine and collagen-coated coverslips. After 24 h of incubation at 37°C, cultures were treated with 1 μM 5-fluoro-5-deoxyuridine (FdU; Sigma) and grown for 14–28 days in supplemented Ultraculture medium prior to examination.

### Quantitative colocalization analysis

Colocalization of NET with plasma membrane and vesicular proteins was achieved on multiple, randomly selected fields of each preparation as previously described [49,53]. Fields from 10–30 per sample were used for each antibody. For axonal distribution analysis, regions of SCG processes were chosen that did not contain varicosities. Intensity values were measured using NIH Image and normalized to the peak intensity value. Paired pixel intensities were plotted as a scatter plot (Prism; GraphPad). The data were used to create the intensity correlation analysis plots and quantified by the intensity correlation quotient [71]. The ICA/ICQ method is based on covariance of signal intensities from two fluorophores in space and is evaluated via the nonparametric sign-test analysis [71]. The normal approximation of the sign test was used to test if these values were significantly different from 0 [71]. Alternatively, data were analyzed by one-sample t-test comparing means versus a value of 0. Multiplying the difference of staining intensity (A) at a particular pixel from the mean intensity (a) for that fluorophore across the analyzed space by the corresponding quantity for the other fluorophore (B-b) at the same pixel yields the Product of the Differences from the Mean [PDM = (A-a)(B-B)] for that pixel. Positive PDM values arise when the staining intensity of both are above the mean or are both present at high values below the mean. ICQ values were calculated first by determining the ratio between the number of positive PDM values and the total number of pixel values. From this ratio, 0.5 is subtracted to yield ICQ values distributed between -0.5 and +0.5 where random colocalization gives an ICQ of ~0, segregated or asynchronous colocalization gives 0 > ICQ ~-0.5, and dependent or synchronous colocalization yields 0 < ICQ ≤ + 0.5 [71]. PDM images were created using a plug-in for ImageJ found at .

## List of Abbreviations

NE: norepinephrine; DA: dopamine; NET: norepinephrine transporter; DAT: dopamine transporter; SERT: serotonin transporter; VMAT: vesicular monoamine transporter; DBH: dopamine beta hydroxylase; SNARE: SNAP receptor; SCG: superior cervical ganglion; BNST: bed nucleus of the stria terminalis; LC: locus ceruleus

## Authors' contributions

HJGM, AG and RDB organized all facets of the research. HJGM performed the immunolocalization in SCG and brain and subcellular fractionation studies in SCG and brain, and with AG and RDB wrote the paper. QH developed the NET-05 monoclonal antibody and performed initial tests of specificity by western blots. AS and DGW performed immunocytochemical studies in brain and assisted in manuscript development. JW helped develop and shared the methodologies for SCG primary culture. JLM helped edit the manuscript. All authors read and approved the final manuscript.
